# The association between pneumococcal vaccination, ethnicity, and the nasopharyngeal microbiota of children in Fiji

**DOI:** 10.1186/s40168-019-0716-4

**Published:** 2019-07-16

**Authors:** Laura K. Boelsen, Eileen M. Dunne, Moana Mika, Stefanie Eggers, Cattram D. Nguyen, F. Tupou Ratu, Fiona M. Russell, E. Kim Mulholland, Markus Hilty, Catherine Satzke

**Affiliations:** 1 Infection and Immunity, Murdoch Children’s Research Institute, Royal Children’s Hospital, Parkville, Victoria Australia; 2 Department of Paediatrics, The University of Melbourne, Parkville, Victoria Australia; 3 Institute for Infectious Diseases, University of Bern, Bern, Switzerland; 4 Translational Genomics Unit, Murdoch Children’s Research Institute, Royal Children’s Hospital, Parkville, Victoria Australia; 5 Ministry of Health and Medical Services, Suva, Fiji; 6 Centre for International Child Health, Department of Paediatrics, The University of Melbourne, Parkville, Victoria Australia; 7 London School of Hygiene & Tropical Medicine, London, UK; 8 Department of Microbiology and Immunology, The University of Melbourne at the Peter Doherty Institute for Infection and Immunity, Parkville, Victoria Australia

**Keywords:** *Streptococcus pneumoniae*, Pneumococcal conjugate vaccination, Nasopharyngeal microbiota, Ethnicity, Fiji

## Abstract

**Background:**

*Streptococcus pneumoniae* is a significant global pathogen that colonises the nasopharynx of healthy children. Pneumococcal conjugate vaccines, which reduce nasopharyngeal colonisation of vaccine-type *S. pneumoniae*, may have broader effects on the nasopharyngeal microbiota; however, data are limited. In Fiji, nasopharyngeal carriage prevalence of *S. pneumoniae* and other colonising species differ between the two main ethnic groups. Here, we examined the association between the 7-valent pneumococcal conjugate vaccine (PCV7) and the nasopharyngeal microbiota of children in Fiji, including for each of the two main ethnic groups—indigenous Fijians (iTaukei) and Fijians of Indian descent (FID).

**Method:**

The nasopharyngeal microbiota of 132 Fijian children was examined using nasopharyngeal swabs collected from 12-month-old iTaukei and FID children who were vaccinated (3 doses PCV7) or unvaccinated in infancy as part of a phase II randomised controlled trial. Microbiota composition was determined by sequencing the V4 region of the 16S rRNA gene. Species-specific carriage of *S. pneumoniae*, *Haemophilus influenzae*, *Moraxella catarrhalis* and *Staphylococcus aureus* was determined using real-time quantitative PCR. Associations between microbiota composition and other host and environmental factors were considered in the analysis.

**Results:**

PCV7 had no overall impact on microbial diversity or composition. However, ethnic differences were observed in both diversity and composition with iTaukei children having higher relative abundance of *Moraxella* (*p* = 0.004) and *Haemophilus* (*p* = 0.004) and lower relative abundance of *Staphylococcus* (*p* = 0.026), *Dolosigranulum* (*p* = 0.004) and *Corynebacterium* (*p* = 0.003) compared with FID children. Further, when we stratified by ethnicity, associations with PCV7 could be detected: vaccinated iTaukei children had a lower relative abundance of *Streptococcus* and *Haemophilus* compared with unvaccinated iTaukei children (*p* = 0.022 and *p* = 0.043, respectively); and vaccinated FID children had a higher relative abundance of *Dolosigranulum* compared with unvaccinated FID children (*p* = 0.037). Children with symptoms of an upper respiratory tract infection (URTI) had a significantly different microbiota composition to children without symptoms. The microbiota composition of iTaukei children without URTI symptoms was most similar to the microbiota composition of FID children with URTI symptoms.

**Conclusions:**

Associations between PCV7 and nasopharyngeal microbiota differed within each ethnic group. This study highlights the influence that ethnicity and URTIs have on nasopharyngeal microbiota.

**Electronic supplementary material:**

The online version of this article (10.1186/s40168-019-0716-4) contains supplementary material, which is available to authorized users.

## Background

*Streptococcus pneumoniae* (the pneumococcus) is a Gram-positive bacterium that causes a range of diseases, including otitis media, pneumonia and meningitis, and is a major cause of morbidity and mortality in children under 5 years of age worldwide [1]. Colonisation of the nasopharynx by *S. pneumoniae* is generally asymptomatic and is considered a precursor for pneumococcal disease [2, 3]. Pneumococcal conjugate vaccines (PCVs) target the pneumococcal capsular polysaccharide of common disease-causing serotypes, with current paediatric vaccine formulations targeting 10 or 13 out of over 90 known serotypes [4]. PCVs provide direct protection against infection and carriage. The reduction in vaccine-type carriage in vaccinees reduces transmission to unvaccinated individuals, thereby resulting in indirect (herd) effects [5–7]. In settings where PCVs have been introduced, serotype replacement has occurred, whereby serotypes not included in PCVs have become more prominent in carriage and disease [8, 9]. There is conflicting evidence about whether species replacement can also occur following pneumococcal vaccination, with mixed evidence for the common respiratory pathogens *Haemophilus influenzae*, *Moraxella catarrhalis* and *Staphylococcus aureus* [10–13]. Interactions with *S. pneumoniae* are thought to underpin vaccine-related changes in prevalence for these pathogens, with a negative interaction between *S. pneumoniae* and *S. aureus*, and generally positive associations between *S. pneumoniae* and *H. influenzae,* and *S. pneumoniae* and *M. catarrhalis* [14–17].

There is some evidence that PCVs may alter nasopharyngeal bacterial composition and diversity; however, findings have not been consistent across the four published studies [18–21]. Biesbroek et al. found that the 7-valent pneumococcal conjugate vaccine (PCV7) was associated with shifts in microbial composition and increases in bacterial diversity in Dutch children at 12, but not at 24, months of age [18]. In contrast, Feazel et al. found the 10-valent pneumococcal conjugate vaccine (PCV10) had no effect on the microbiome of Kenyan children (aged 12–59 months) 180 days after vaccination [19]. Two studies have been done in Swiss children. In children < 2 years of age with acute otitis media, PCV7 reduced the prevalence of commensal families [20]. The other study in healthy infants over the first year of life found that children vaccinated in the 13-valent pneumococcal conjugate vaccine (PCV13) era had higher microbial diversity and microbiota stability than children vaccinated in the PCV7 era; this was likely associated with the lower pneumococcal carriage prevalence in the PCV13 era [21].

Carriage prevalence of bacterial species can vary by geographic region and socio-economic status [22, 23]. Children in low- and middle-income countries, and susceptible populations in high-income settings, often have a high prevalence of pneumococcal colonisation, suggesting that the impact of vaccination on the microbiome could be different in these settings compared with children in developed countries [22].

There are two main ethnic groups in Fiji, indigenous Fijians (iTaukei) and Fijians of Indian descent (FID). The iTaukei have higher carriage prevalence of *S. pneumoniae* and higher burden of pneumococcal disease compared with FID [24, 25]. Previously, we conducted the Fiji Pneumococcal Project (FiPP), a randomised phase II vaccine trial that evaluated various schedules of PCV7 followed by the 23-valent pneumococcal polysaccharide vaccine as a booster at 12 months of age [26–29]. Results from FiPP at 17 months of age, and a long-term follow-up to the study, highlighted differences in carriage prevalence of *S. pneumoniae*, *S. aureus*, *H. influenzae* and *M. catarrhalis* between the two main ethnic groups [25, 30], suggesting they have distinct microbial profiles. We also found that the 23-valent pneumococcal polysaccharide vaccine given at 12 months of age had differential effects on long-term carriage of *S. pneumoniae* and *S*. *aureus* for the two ethnic groups [30].

Therefore, we hypothesise that PCVs may have differential effects on microbiota depending on the ethnicity of the child. To examine this directly, we used samples collected as part of the FiPP to determine the association between PCV and nasopharyngeal microbiota in Fijian children aged 12 months, including specific associations within each ethnic group.

## Results

Overall, 144 samples (36 samples each from unvaccinated iTaukei children, PCV7 vaccinated iTaukei children, unvaccinated FID children and PCV7 vaccinated FID children), 4 sample repeats, 7 extraction controls and 5 PCR (no template) controls were sequenced resulting in a total of 62,038,435 sequence reads and an average of 387,740 reads per sample. Following sequence processing, 59% (36,344,642 total; 227,154 per sample) of these sequence reads remained for analysis. Twelve samples were excluded because they shared greater than 20% sequence read similarity with controls (*n* = 11) or had fewer than 50,000 sequence reads (*n* = 1). In the remaining 132 samples, we identified 4036 operational taxonomic units (OTUs) in total and 847 OTUs with unique taxonomic names. The most common genera (*Dolosigranulum*, *Pseudomonas*, *Corynebacterium*, *Moraxella*, *Haemophilus*, *Streptococcus* and *Staphylococcus*) accounted for at least 90% of all sequence reads in 118 out of the 132 (89%) of samples.

### Study participant characteristics

Participant characteristics by ethnicity and vaccination status are shown in Table 1. There were few differences between unvaccinated and vaccinated children within each ethnic group, although among FID children, there were fewer males in the unvaccinated group compared with the vaccinated group and vaccinated children were slightly heavier. There was some evidence that fewer vaccinated FID children used antibiotics in the previous 2 weeks compared with unvaccinated FID children (0% vs. 13%). There was also some evidence that a greater number of unvaccinated iTaukei children were breastfed at the time of swab collection compared with vaccinated iTaukei children (79% vs. 56%). Pooled participant characteristics by both vaccination status and by ethnicity were also considered (see Additional file 1: Tables S1 and S2), with a higher proportion of unvaccinated children breastfed compared with vaccinated children (74% vs. 54%, *p* = 0.019). Mean weight was higher in iTaukei children compared with FID children (5023 g vs. 4382 g, *p* < 0.001) and more iTaukei children were exposed to cigarette smoke than FID children (55% vs. 18%, *p* = 0.038).

**Table 1:** Participant characteristics for children aged 12 months by PCV vaccination status within each ethnic group

Characteristics at swab collection	No PCV7 iTaukei (*n* = 33)	PCV7 iTaukei (*n* = 34)	*p* value	No PCV7 FID (*n* = 32)	PCV7 FID (*n* = 33)	*p* value
Male, *n* (%)	18 (55)	19 (56)	1.000	*12 (38)*	*22 (67)*	0.026
Swab collected in wet season^1^, *n* (%)	25 (76)	28 (82)	0.560	25 (78)	20 (61)	0.180
Mean weight^2^ in g (SD)	4998 (584)	5084 (579)	0.658	*4243 (547)*	*4566 (443)*	0.018
Breastfeeding, *n* (%)	26 (79)	19 (56)	0.068	22 (69)	17 (52)	0.208
Median age breastfeeding stopped^3^ in wks (IQR)	26 (26-40)	28 (18-35)	0.523	9 (2-31)	27 (15-40)	0.086
Exposure to cigarette smoking, *n* (%)	21 (64)	16 (47)	0.222	12 (38)	12 (36)	1.000
Prior antimicrobial use^4^, *n* (%)	4 (12)	5 (15)	1.000	4 (13)	0 (0)	0.053
Symptoms of URTI [any], *n* (%)	11 (33)	14 (41)	0.615	8 (25)	6 (18)	0.558
Runny nose, *n* (%)	10 (30)	9 (26)	0.791	6 (19)	5 (15)	0.751
Cough, *n* (%)	7 (21)	10 (29)	0.576	4 (13)	5 (15)	1.000

*URTI* upper respiratory tract infection, *SD* standard deviation, *iTaukei* indigenous Fijian, *FID* Fijian of Indian Descent, Statistically significant differences (*p* < 0.05) as calculated by Fisher’s Exact test are shown in italics.

^1^Wet season = November–April

^2^Weight data were incomplete for vaccinated children—13/34 available for iTaukei children, 24/33 available for FID children

^3^Data only included for children that had stopped breastfeeding

^4^Antimicrobial use in the prior two weeks as reported by parent/guardian

### Overall association between PCV7 and nasopharyngeal microbiota

**Fig. 1:**
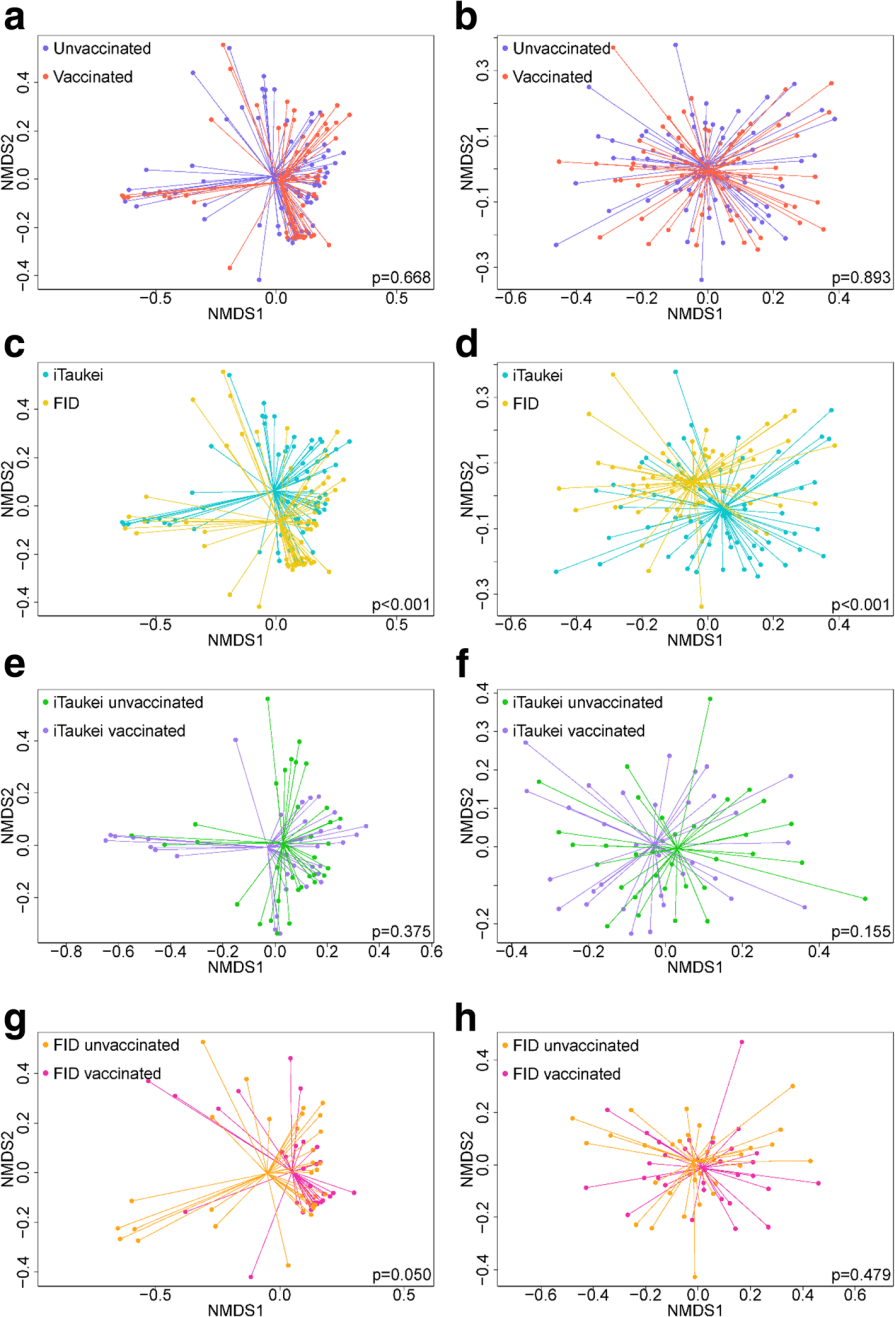
nMDS of the dissimilarity in microbial composition using both abundance-based and binary-based measures by vaccination status (**a** and **b**, respectively), by ethnicity (**c** and **d**, respectively), in iTaukei children by vaccination status (**e** and **f**, respectively) and in FID children by vaccination status (**g** and **h**, respectively). The centre points represent the mean of each group and the lines represent distance from the mean for each sample. *p* values calculated using PERMANOVA. Stress values for figures are 0.158 (**a** and **c**), 0.216 (**b** and **d**), 0.154 (**e**), 0.218 (**f**), 0.149 (**g**) and 0.215 (**h**)

PCV7 vaccination had no significant association with the richness of nasopharyngeal microbiota (median number of OTUs in unvaccinated children 84 vs. 62 in vaccinated children, *p* = 0.137) or Shannon diversity (median of 1.36 in unvaccinated children vs. 1.28 in vaccinated children, *p* = 0.115) (see Additional file 2: Figure S1a and S1b). We explored differences in overall microbial composition between unvaccinated and vaccinated children, using Jaccard dissimilarity (both abundance-based and binary-based) and using non-metric multidimensional scaling (nMDS) plots as an ordination method. Overall, vaccination appeared to have little impact on the microbial composition of the nasopharynx regardless of whether abundance-based (Fig. 1a, *p* = 0.668) or binary-based (Fig. 1b, *p* = 0.893) dissimilarity was considered. Looking specifically at the seven most common genera (Table 2), PCV7 was only associated with changes to the relative abundance of *Streptococcus* (*p* = 0.030), with a lower relative abundance of *Streptococcus* in vaccinated children (median 0.59%) compared with unvaccinated children (median 1.02%). The random forest model considering the association between PCV7 vaccination and microbial composition was not significant (*p* = 0.836). As the 16S sequencing analysis was generally limited to genus level, we examined carriage prevalence and density of the common respiratory pathogens *S. pneumoniae*, *H. influenzae*, *M. catarrhalis* and *S. aureus* using species-specific real-time quantitative PCR (qPCR), finding no differences between unvaccinated children and vaccinated children (Additional file 3:Figure S2).

**Table 2:** Per cent median relative abundance (IQR) for the seven most common genera by vaccination status (overall), by ethnicity, and by vaccination status within each ethnic group

	No PCV7 (*n* = 65)	PCV7 (*n* = 67)	*p* value^1^	iTaukei (*n* = 67)	FID (*n* = 65)	*p* value^1^	iTaukei			FID		
							No PCV7 (*n* = 33)	PCV7 (*n* = 34)	*p* value^1^	No PCV7 (*n* = 32)	PCV7 (*n* = 33)	*p* value^1^
*Pseudomonas* ^2^	0.04 (0.01–0.50)	0.03 (0.01–0.24)	0.212	0.04 (0.01–0.44)	0.04 (0.02–0.27)	0.122	0.04 (0.01–0.16)	0.03 (0.01–0.77)	0.208	0.06 (0.02–0.58)	0.03 (0.02–1.00)	0.090
*Moraxella*	28.73 (8.55–44.56)	33.19 (0.30–50.78)	0.823	*29.33* *(1.97–46.70)*	*0.20* *(0.05–30.67)*	*0.004*	28.73 (8.55–44.56)	33.19 (0.30–50.78)	0.927	0.11 (0.04–29.14)	1.44 (0.05–30.78)	0.690
*Staphylococcus* ^2^	0.02 (0.01–0.16)	0.02 (0.01–0.05)	0.887	*0.02* *(0.01–0.06)*	*0.06* *(0.02–0.26)*	*0.026*	0.02 (0.01–0.16)	0.02 (0.01–0.05)	0.738	0.06 (0.02–0.26)	0.06 (0.03–0.27)	0.403
*Dolosigranulum*	21.26 (6.07–32.46)	15.94 (4.80–36.53)	0.777	*18.18* *(6.02–32.93)*	*36.42* *(13.80–51.38)*	*0.004*	21.26 (6.07–32.46)	15.94 (4.80–36.53)	0.632	*29.25* *(10.35–49.37)*	*44.59* *(30.62–55.11)*	*0.037*
*Streptococcus* ^2^	*1.02* *(0.16–7.15)*	*0.59* *(0.16–1.86)*	*0.030*	0.88 (0.23–4.72)	0.62 (0.12–3.42)	0.085	*2.02* *(0.48–7.18)*	*0.52* *(0.14–1.78)*	*0.022*	0.56 (0.09–5.46)	0.62 (0.21–1.89)	0.438
*Corynebacterium*	6.95 (0.99–17.98)	3.71 (1.24–15.99)	0.916	*4.83* *(1.22–17.08)*	*17.92* *(3.42–37.60)*	*0.003*	6.95 (0.99–17.98)	3.71 (1.24–15.99)	0.735	10.39 (3.06–33.35)	24.05 (6.03–42.25)	0.314
*Haemophilus* ^2^	1.02 (0.04–8.32)	0.11 (0.02–9.33)	0.053	*0.59* *(0.03–9.17)*	*0.03* *(0.01–0.39)*	*0.004*	*1.02* *(0.04–8.32)*	*0.11* *(0.02–9.33)*	*0.043*	0.03 (0.01–0.30)	0.04 (0.01–0.86)	0.462

Data are median (interquartile range)

^1^*p* value calculated following multivariable linear regression adjusting for ethnicity, symptoms of an upper respiratory tract infection, exposure to household cigarette smoke, breastfeeding status, year of swab collection, season of swab collection, antibiotic use in the previous two weeks and sex of the child. An interaction between vaccination status and ethnicity was included in models for *Pseudomonas* (*p* = 0.026), *Dolosigranulum* (*p* = 0.093), *Streptococcus* (*p* = 0.033) and *Haemophilus* (*p* = 0.029) but not in models for *Moraxella* (*p* = 0.945), *Corynebacterium* (*p* = 0.387) and *Staphylococcus* (*p* = 0.453)

^2^Log transformation of relative abundance was used in the linear regression models

### Ethnic differences

**Fig. 2:**
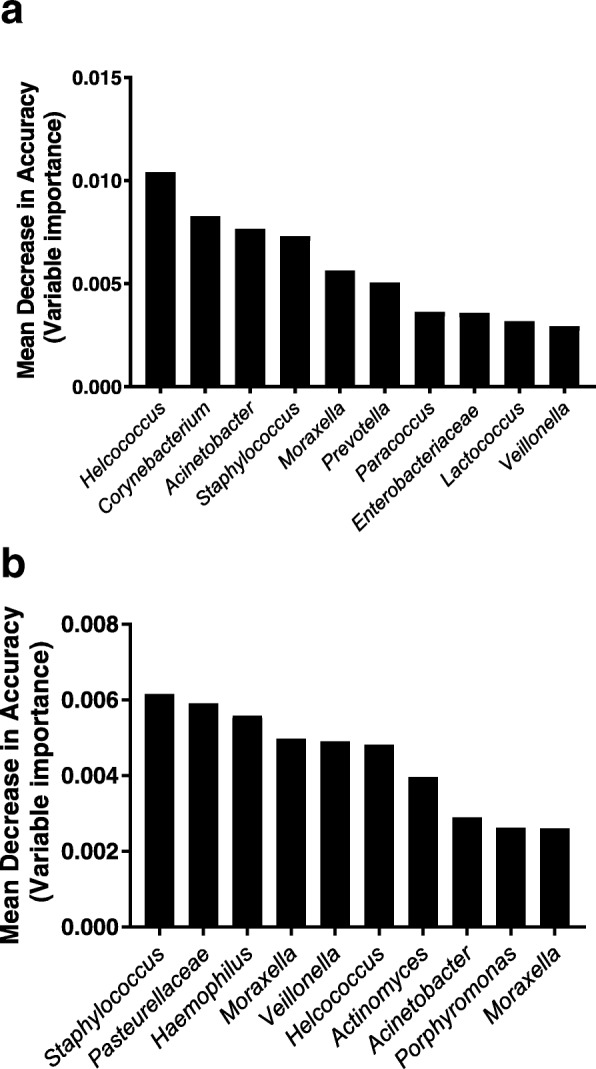
Top ten most important OTUs (as ranked by mean decrease in accuracy) in random forest models for ethnicity (**a**) and symptoms of an URTI (**b**). Only ten OTUs are shown to highlight the most significant OTUs that separate iTaukei and FID children, and children with and without symptoms of an URTI

We next examined difference in nasopharyngeal microbiota by ethnic group. While the ethnicity of a child did not influence OTU richness (median number of OTUs in iTaukei children of 68 vs. 72 in FID children, *p* = 0.436), it did have an influence on Shannon diversity. OTUs were more evenly spread in iTaukei children compared with FID children (median Shannon diversity index of 1.33 vs. 1.07, *p* = 0.006) (see Additional file 2: Figure S1c and S1d). The overall microbial composition of the nasopharynx was significantly different between iTaukei and FID children using both abundance-based (*p* < 0.001) and binary-based measures (*p* < 0.001) (Fig.1c and d). Of the most common genera (Table 2), iTaukei children had higher relative abundance of *Moraxella* and *Haemophilus* and lower relative abundance of *Staphylococcus*, *Dolosigranulum* and *Corynebacterium*. The random forest model also showed an association between ethnicity and microbial composition (*p* < 0.001). The most important OTUs in the random forest model included *Corynebacterium*, *Staphylococcus* and *Moraxella*, as well as less abundant OTUs *Helcococcus*, *Acinetobacter* and *Prevotella* (Fig. 2a). This *Helcococcus* OTU was more prevalent in iTaukei children compared with FID children (78% in iTaukei vs. 52% in FID children, *p* = 0.003) and was also present at a higher relative abundance (median relative abundance of 0.4% in iTaukei children vs. 0.001% in FID children, *p* < 0.001). By species-specific qPCR, the carriage prevalence of *S. pneumoniae*, *H. influenzae* and *M. catarrhalis* was higher in iTaukei children than FID children (69% vs. 26%, 69% vs. 28% and 91% vs. 37%, respectively, all *p* < 0.001). The carriage prevalence of *S. aureus* was similarly low in iTaukei children (4%) and FID children (6%). No differences in species-specific density between ethnic groups were observed (Additional file 3: Figure S3).

### Association between PCV7 and nasopharyngeal microbiota within each ethnic group

Given the differences in microbiota composition between ethnic groups, we examined the association between PCV7 and nasopharyngeal microbiota after stratifying by ethnicity. There were no significant differences in OTU richness or Shannon diversity between unvaccinated and vaccinated children within either ethnic group (iTaukei children: median of 84 OTUs and 1.36 Shannon diversity in unvaccinated children vs. 62 OTUs and 1.28 Shannon diversity in vaccinated children, *p* = 0.098 and 0.115, respectively; FID children: median of 70 OTUs and 1.11 Shannon diversity in unvaccinated children vs. 71 OTUs and 1.00 Shannon diversity in vaccinated children, *p* = 0.095 and 0.662, respectively) (see Additional file 2: Figure S1e and S1f). nMDS analyses suggested PCV7 had little association with the nasopharyngeal microbiota of iTaukei children using both abundance-based (*p* = 0.375) and binary-based (*p* = 0.155) measures (Fig. 1e and f). There was some evidence that PCV7 was associated with nasopharyngeal microbial composition of FID children using an abundance-based measure (*p* = 0.05) but not a binary-based measure (*p* = 0.479) (Fig. 1g and h), suggesting that PCV7 may impact the relative abundance, but not the presence, of OTUs in FID children. Random forest models showed no association between microbial composition and vaccination within either ethnic group (*p* = 0.952 in Taukei children and *p* = 0.512 in FID children).

In iTaukei children, unvaccinated children had higher relative abundance of *Streptococcus* compared with vaccinated children (Table 2). Similarly, unvaccinated iTaukei children had a higher relative abundance of *Haemophilus* compared with vaccinated iTaukei children. No other differences in the most common genera between unvaccinated and vaccinated iTaukei children were seen.

In FID children, those that were unvaccinated had lower relative abundance of *Dolosigranulum* compared with children who received PCV7 (Table 2). No other PCV7-associated differences in common genera were observed in FID children.

Using species-specific qPCR assays, we examined carriage prevalence and density within each ethnic group for *S. pneumoniae*, *H. influenzae*, *M. catarrhalis* and *S. aureus* comparing unvaccinated and vaccinated children (Additional file 3: Figure S4). Within each ethnic group, there were no significant differences in carriage prevalence or density by vaccination status for any of the four bacterial species examined.

### Symptoms of an upper respiratory tract infection

We examined the association between other factors (seasonality, exposure to cigarette smoke, antibiotic use in the previous 2 weeks, year of swab collection, sex, current breastfeeding status and symptoms of an upper respiratory tract infection (URTI)—either cough or runny nose) and overall microbiota composition (Additional file 1: Table S1). Only the presence of symptoms of an URTI had a significant impact on microbial composition. In the random forest model for URTI symptoms (*p* = 0.012), the most important OTUs were *Staphylococcus*, an unclassified Pasteurellaceae OTU, *Haemophilus*, *Moraxella*, *Veillonella* and *Helcococcus* (Fig. 2b). Compared with children without URTI symptoms, children with URTI symptoms had higher relative abundance of *Moraxella*, *Haemophilus* and *Streptococcus* and lower relative abundance of *Dolosigranulum* and *Corynebacterium* (Table 3).

**Table 3:** Median relative abundance (%) for the seven most common genera by symptoms of an upper respiratory tract infection (URTI)

	No URTI (*n* = 65)	URTI (*n* = 67)	*p* value^1^
*Pseudomonas* ^2^	0.04 (0.01–0.49)	0.04 (0.01–0.14)	0.348
*Moraxella*	*7.51* *(0.06–37.94)*	*30.55* *(0.31–48.14)*	*0.044*
*Staphylococcus* ^2^	0.04 (0.01–0.17)	0.02 (0.01–0.17)	0.458
*Dolosigranulum*	*32.75* *(9.46–48.89)*	*15.08* *(4.25–32.09)*	*0.044*
*Streptococcus* ^2^	*0.55* *(0.12–3.41)*	*1.57* *(0.29–7.57)*	*0.040*
*Corynebacterium*	*13.54* *(3.53–34.10)*	*2.61* *(0.80–14.55)*	*0.039*
*Haemophilus* ^2^	*0.04* *(0.01–2.78)*	*0.81* *(0.09–16.48)*	*0.002*

Data are median (interquartile range)

^1^*p* value calculated following multivariable linear regression adjusting for ethnicity, presence of URTI symptoms, exposure to household cigarette smoke, breastfeeding status, year of swab collection, season of swab collection, antibiotic use in the previous 2 weeks and sex. An interaction between vaccination status and ethnicity was included in models for *Pseudomonas* (*p* = 0.026), *Dolosigranulum* (*p* = 0.093), *Streptococcus* (*p* = 0.033) and *Haemophilus* (*p* = 0.029), but not in models for *Moraxella* (*p* = 0.945), *Corynebacterium* (*p* = 0.387) and *Staphylococcus* (*p* = 0.453)

^2^Log transformation of relative abundance was used in the linear regression models

**Fig. 3:**
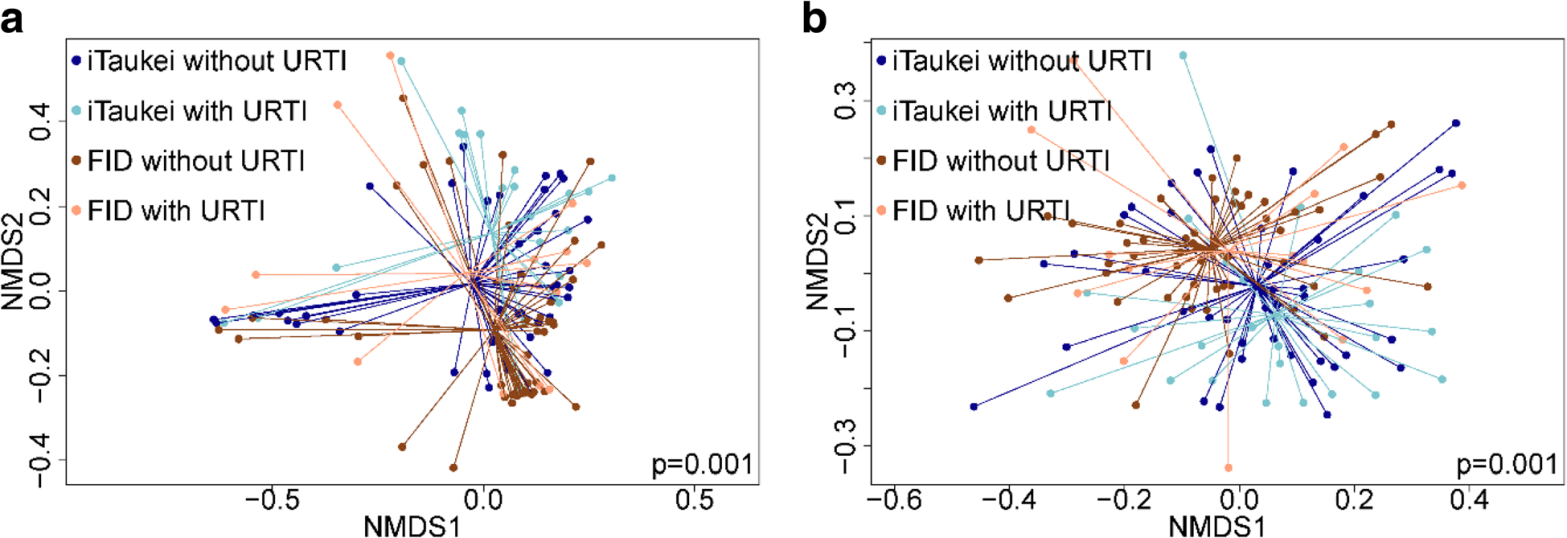
nMDS of the dissimilarity in microbial composition by ethnicity and symptoms of an upper respiratory tract infection using abundance-based (**a**) and binary-based (**b**) measures. Shown are iTaukei children, with (light blue) or without (dark blue) symptoms of an upper respiratory tract infection, and FID children, with (light brown) or without (dark brown) symptoms of an upper respiratory tract infection. The centre points represent the mean of each group and the lines represent distance from the mean for each sample. There was a significant difference between the four groups (*p* = 0.001), *p* value calculated using PERMANOVA. Stress values for figures are 0.158 (**a**) and 0.216 (**b**)

Interestingly, not only did children with symptoms of an URTI have significantly different microbiota compared with children without symptoms, but the microbiota composition of iTaukei children without URTI symptoms was most similar to the microbiota composition of FID children with URTI symptoms (Fig. 3). When analysis of the most common genera was stratified by ethnicity, the relative abundance in *Haemophilus* was higher in iTaukei children with URTI symptoms compared to those without symptoms (Additional file 1: Table S2). No other significant differences were observed, but trends were consistent in both ethnic groups and with the overall results shown in Table 3.

### Microbial composition and dominant taxa

**Fig. 4:**
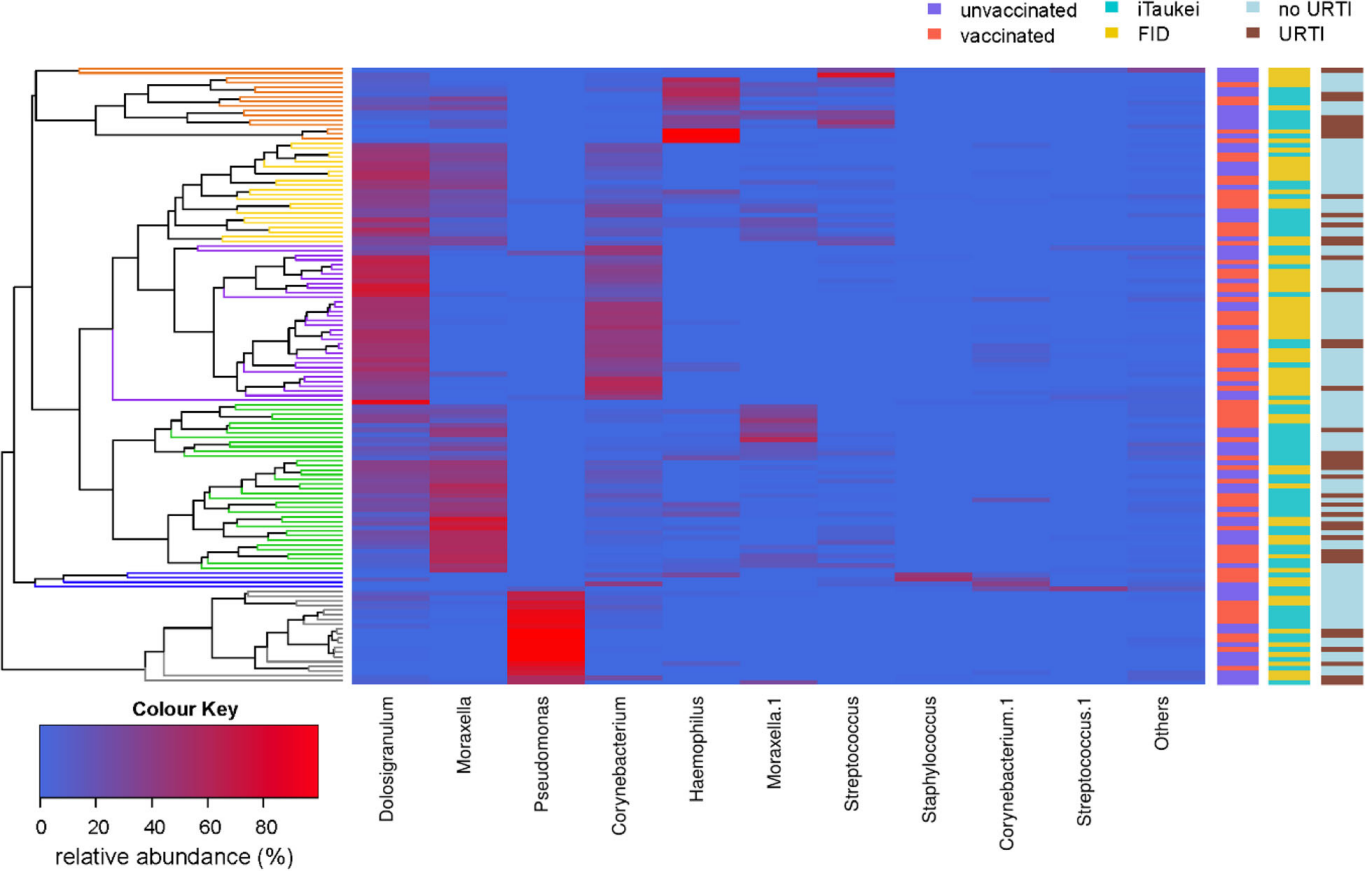
Relative abundance (%) heatmap including all 132 samples. Sample clustering is shown on the left-hand side. Taxa with a relative abundance above 30% in at least one sample are shown at the bottom. Where multiple OTUs are from the same genus ‘.1’ has been used for subsequent OTUs. ‘Others’ represents the remaining taxa in each sample. Clusters for samples have been coloured to show clustered groups: Cluster 1 (shown in dark blue), Cluster 2 (shown in green) dominated by *Moraxella*, Cluster 3 (shown in yellow) dominated by *Dolosigranulum*, Cluster 4 (shown in purple) dominated by *Dolosigranulum* and *Corynebacterium*, Cluster 5 (shown in orange) dominated by *Haemophilus*, and Cluster 6 (shown in grey) dominated by *Pseudomonas*. The vaccination status (unvaccinated—blue; vaccinated—red), ethnicity (iTaukei—turquoise; FID—gold) and whether the child had any symptoms of an upper respiratory tract infection (no URTI—light blue; URTI—brown) are shown in bars on the right of the heatmap

We next looked at microbial composition in the samples by generating a heatmap and clustering samples based on their composition using a Jaccard distance matrix (Fig. 4). Most samples were dominated by one or two taxa with the following clusters identified by their dominant taxa (Fig. 3): Cluster 2 (*n* = 36) dominated by *Moraxella*; Cluster 3 (*n* = 22) dominated by *Dolosigranulum*; Cluster 4 (*n* = 34) dominated by *Dolosigranulum*; Cluster 5 (*n* = 16) dominated by *Haemophilus* and *Corynebacterium*; and Cluster 6 (*n* = 20) dominated by *Pseudomonas*. Cluster 1 contained four samples that did not fit into any of the other clusters.

Associations between clusters and host factors were considered, with several clusters associated with ethnicity and symptoms of an URTI. Compared with iTaukei children, FID children were more likely to have a cluster 4 microbiota profile (40% FID children vs. 12% iTaukei children, *p* < 0.001) and less likely to have a cluster 2 microbiota profile (17% FID children vs. 37% iTaukei children, *p* = 0.011). Cluster 4 was associated with a higher proportion of children without URTI symptoms (31% no URTI children vs. 13% URTI children, *p* = 0.030). There was also some evidence that cluster 5 and cluster 2 were associated with symptoms of an URTI (9% no URTI children vs. 21% URTI children, *p* = 0.078; and 23% no URTI children vs. 38% URTI children, *p* = 0.086, respectively).

Owing to concern that *Pseudomonas* was a potential contaminant, we cultured eight samples where *Pseudomonas* comprised of > 90% of sequence reads. We detected *Pseudomonas* on selective agar for six of the eight samples (two samples had no growth), which were identified as *P. fluorescens* (*n* = 3) and *P. fragi* (*n* = 3) by MALDI-TOF MS.

### Bacterial interactions

**Fig. 5:**
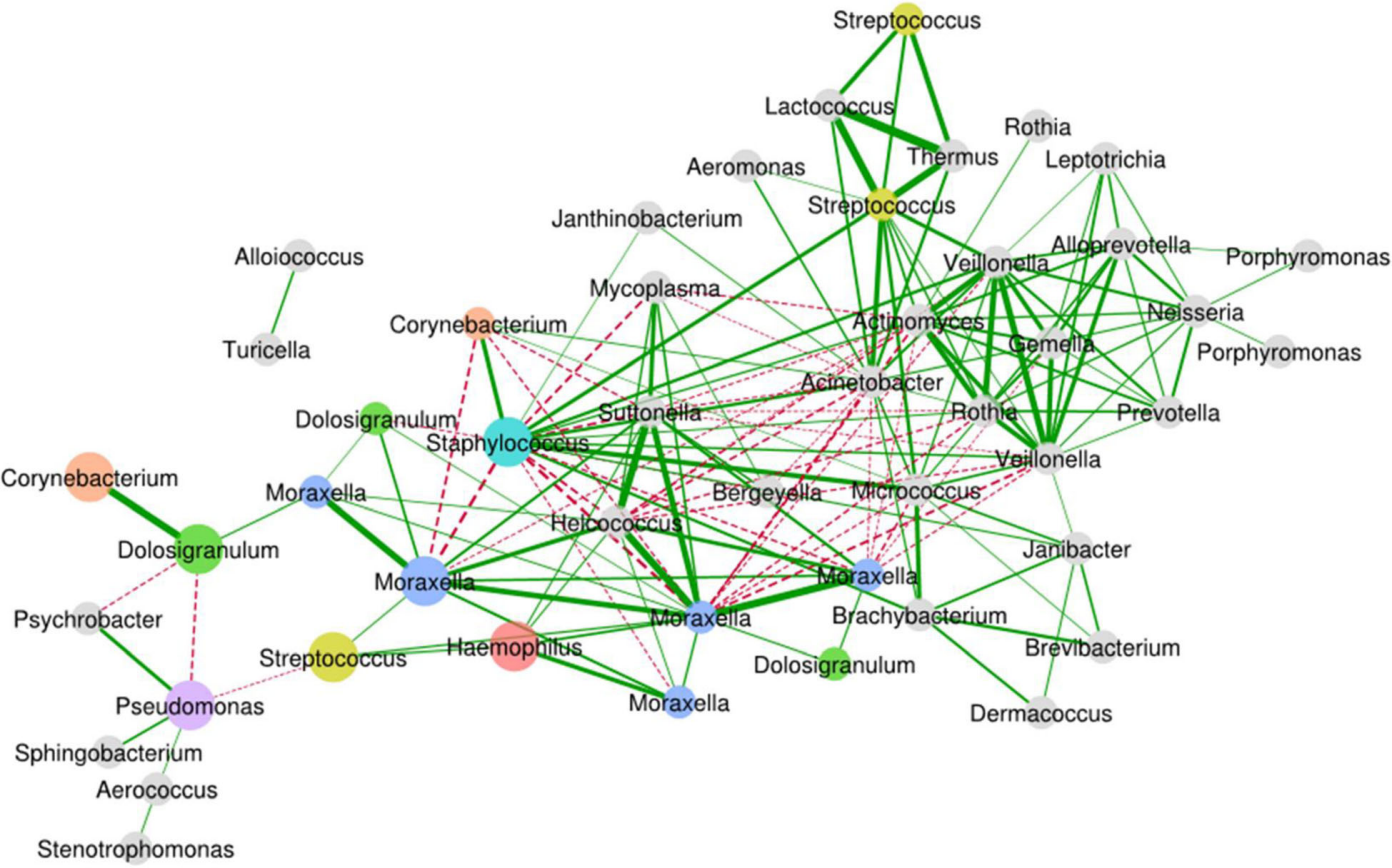
Network correlation map based on the 100 most common OTUs considering samples from all 132 children. Shown are SparCC correlations with *p* value < 0.05 and *r* > 0.3. The main OTUs from the top seven genera are shown by larger node size. Colours of nodes are grey except for the seven most common genera. The edge (connecting line) colour represents a positive (green solid lines) or negative (red dashed lines) correlation between two OTUs, with thicker lines representing stronger correlations. Only those correlations with genus-level classification are shown and are labelled with the OTU genus

We examined the relationships between the 100 most common OTUs using the network inference tool, SparCC (Fig. 5). Of particular note for the correlation network, the predominant *Streptococcus* OTU had positive relationships with two of the *Moraxella* OTUs (*r* = 0.32, *p* < 0.001 and *r* = 0.34, *p* < 0.001) and the predominant *Haemophilus* OTU (*r* = 0.36, *p* < 0.001); and a negative relationship with *Pseudomonas* (*r* = 0.31, *p* < 0.001). There was no significant interaction between *Streptococcus* and *Dolosigranulum* (*r* = 0.03, *p* = 0.379).

## Discussion

There are limited data on the impact of pneumococcal vaccination on respiratory microbiota, particularly from children in low- and middle-income countries. We investigated the association between pneumococcal vaccination and nasopharyngeal microbiota in 12-month-old Fijian children approximately 8.5 months after their third dose of PCV7. Given that the two ethnic groups in Fiji have marked differences in pneumococcal carriage and disease prevalence, we examined the associations with pneumococcal vaccination by ethnicity.

We found little evidence of a broader association between PCV7 and nasopharyngeal microbiota, beyond reducing the relative abundance of *Streptococcus* in vaccinated children compared with unvaccinated children. Our findings were consistent with a study in Kenyan children aged 12–59 months, where no differences were observed 4 months after vaccination [19]. Some effects of pneumococcal vaccination have been observed in studies in Swiss children with otitis media aged < 2 years, where vaccination reduced commensals such as Streptococcaeae (excluding *pneumococci*) and Corynebacteriaeae [20], and in Dutch children aged 12 months, where vaccinated children had increased absolute abundance of *Haemophilus* and *Staphylococcus* as well as *Actinomyces*, *Rothia*, *Neisseria* and *Veillonella* [18]. There are some notable differences between these studies and ours, particularly with regard to vaccine formulation and schedule and age at which microbiota was assessed. These differences include the third dose of PCV in our study given to children at 14 weeks of age compared to 11 months of age in the Netherlands study and the use of the 10-valent PCV in the Kenyan study rather than 7-valent PCV used in our study. Additionally, in some cases, the laboratory methods (such as the sequencing platform, the 16S rRNA region sequenced and the DNA extraction method used) differed between studies which may contribute to some differences in findings. Sequencing depth also varies between studies, and some studies use a subsampling approach for analysis. We conducted an exploratory analysis using subsampling and found no observable differences (data not shown) and therefore did not use subsampling in our analyses. We cannot exclude the possibility that other methodological approaches, such as the use of other DNA extraction methods, would yield slightly different results. However, available data suggests that any PCV effects on nasopharyngeal microbiota may occur soon after vaccination and may be transient.

Our previous studies in Fiji [25, 30] found significant differences in bacterial carriage between iTaukei and FID children but only examined a limited number of bacterial species. Here, we found substantial differences in the entire nasopharyngeal microbiota based on ethnicity. Ethnicity influenced both microbial community structure, with higher Shannon diversity in iTaukei children, and community composition, with differences in the relative abundance of five of the seven most common genera. Of the participant characteristics collected, only weight and exposure to household cigarette smoking differed between the two ethnic groups, and in this study neither of these had a significant association with microbial composition. There are several genetic, environmental and social factors which may contribute to the observed ethnic differences in the microbiota [31]. Differences in genes associated with immunity, such as beta-defensin and pattern recognition receptors have been associated with differences in the nasopharyngeal microbiota and susceptibility to bacterial infections [32, 33]. Polymorphisms, particularly in immunity-related genes, have been linked to microbial community composition, and patterns of polymorphisms have shown to differ by geographic and ethnic origin [34, 35]. There is some evidence that *Helcococcus*, highlighted as one of the most significant OTUs in the random forest model for ethnicity, is associated with the presence of chemokine CXL8 [36], supporting a role for immune factors in the observed ethnic differences.

The 2004 Fiji National Nutrition survey revealed several dietary and lifestyle factors that differed between the two ethnic groups including differences in consumption of white meat (e.g. chicken) and preserved foods, as well as levels of physical activity and cigarette use [37]. There is some evidence that diet and composition of intestinal microbiota may be determinants of respiratory microbiota [38, 39]; however, information on diet (apart from breastfeeding status) was not collected in our study.

Other household characteristics like social contact and/or environmental factors may also be important in Fiji. Recently, we undertook a social contact survey in Fiji, finding that iTaukei children have a greater frequency of physical contacts than FID children [40]. There is evidence that social contact can influence microbiota [41] but the relative importance of social contact compared with other factors as a determinant of microbiota is unknown. In Fiji, the two main ethnic groups have similar household income [30] so it is unlikely that socio-economic status, a known risk factor for pneumococcal carriage [23], contributes to the observed differences in nasopharyngeal bacteria between iTaukei and FID children. Given the complexity and interconnected nature of factors that can determine microbiota, it is likely multiple factors are involved in the underlying differences in microbial composition between ethnic groups in Fiji.

We next examined the associations with PCV7 after stratifying by ethnicity. In iTaukei children, PCV7 vaccination was associated with reduced relative abundance of both *Streptococcus* and *Haemophilus*, without associations with other common genera. We noted a positive relationship between *Streptococcus* and *Haemophilus,* which may underpin an indirect reduction in *Haemophilus* (i.e. through a direct vaccine-induced reduction in *Streptococcus*). However, there was no evidence that vaccination reduced the carriage prevalence or density of *S. pneumoniae* or *H. influenzae*. While PCVs reduce carriage prevalence of vaccine serotype *S. pneumoniae*, they generally have no effect on overall *S. pneumoniae* carriage prevalence due to serotype replacement by non-vaccine serotypes [42, 43], and so the lack of an overall association in this study with *S. pneumoniae* carriage prevalence is not unexpected. We also saw no association between PCV7 and *S. pneumoniae* carriage density despite the observed difference in relative abundance of *Streptococcus*. However, as the density of *S. pneumoniae* was an absolute measure rather than relative, it is possible that differences in absolute abundance between unvaccinated and vaccinated children may explain the discrepancy between the two sets of results. Consistent with this, Bisbroek et al. [18] found that the absolute abundance of total microbiota was much higher in vaccinated children compared with controls at 24 months of age.

In contrast to the effect in iTaukei children, PCV7 was not associated with changes to the relative abundance of *Streptococcus*, nor *S. pneumoniae* carriage prevalence or density, in FID children. However, the relative abundance of *Dolosigranulum* was significantly higher in vaccinated FID children compared with unvaccinated FID children. *Dolosigranulum* has been reported to have a negative relationship with *S. pneumoniae* [44], and as such, although we noted no association between PCV7 and *Streptococcus* and no direct interaction between *Streptococcus* and *Dolosigranulum*, our findings may have been a result of earlier interactions not captured in our sampling. For example, it is plausible that pneumococcal vaccination may have had a transient effect on *Streptococcus* directly after vaccine administration, which may also have indirectly increased the relative abundance of *Dolosigranulum*, in vaccinated FID children. Microbiota profiles containing *Dolosigranulum* are more stable than those containing *Streptococcus* [45], and therefore it is possible that an effect may have been maintained for *Dolosigranulum* but not *Streptococcus*.

The presence of URTI symptoms had a significant association with nasopharyngeal microbiota. In children with symptoms of an URTI, the relative abundances of *Streptococcus*, *Moraxella* and *Haemophilus* was higher and the relative abundance of *Dolosigranulum* and *Corynebacterium* was lower, compared with children without URTI symptoms. Interestingly, when we looked at community composition by ethnicity and URTI symptoms, we found that the microbiota of FID children with URTI symptoms was similar to the microbiota of iTaukei children without URTI symptoms, although the presence of URTI symptoms shifted microbiota profiles in a similar direction for both ethnic groups. The association with URTI symptoms and *Moraxella* and *Haemophilus* is perhaps unsurprising given that there are known respiratory pathogens within the genera which have been linked to URTIs [46, 47]. *Dolosigranulum* and *Corynebacterium* have been linked with a healthy nasopharyngeal microbiome, and the presence of *Dolosigranulum* and *Corynebacterium* have been associated with a decreased risk of otitis media [44] as well as decreased episodes of respiratory tract infections [45, 48].

A limitation of this study is that viruses were not examined, and URTI symptoms may be related to viral infection. De Steenhuijsen Piters et al. [49] found that respiratory syncytial virus infection and hospitalisation was associated with *Streptococcus* and *Haemophilus* in the nasopharyngeal microbiota in children < 2 years of age. In adults, rhinovirus decreased α-diversity and altered the relative abundance of the genera *Neisseria* and *Propionibacterium* [50].

Contamination can be a significant problem in microbiome studies, particularly for low biomass samples [51]. We sequenced all the PCR products from the extraction and PCR controls, as despite taking several measures to reduce contamination and repeating PCRs with no template added, a PCR product was present in all our controls. Contamination is difficult to eliminate in microbiome studies due to the presence of bacterial components in purified water, DNA extraction kits and PCR reagents [51, 52]. The OTU composition of the controls was substantially different to the samples included in our analysis, giving confidence in our findings. Furthermore, most of the abundant genera in our study have been reported as predominant genera in other studies [20, 53]. The exception is *Pseudomonas,* which is not commonly found in the nasopharynx, but has been identified as a potential contaminant in several studies [51, 54]. In our study, when *Pseudomonas* was found, it was generally present at a high relative abundance in participant samples and at a low relative abundance in the ‘typical’ control profile (< 1% in 10 out 12 controls). Samples containing *Pseudomonas* were distributed across all seven rounds of DNA extractions. We consider it unlikely that *Pseudomonas* is a contaminant in our study, as viable *Pseudomonas* from two different species were cultured from six out of eight samples for which *Pseudomonas* accounted for > 90% of the relative abundance. Nevertheless, when we removed these eight samples from our analysis it did not affect the conclusions of our study. Additionally, given that for other *Pseudomonas* species such as *P. aeruginosa* acquisition increases in warmer seasons and tropical climates [55, 56], it is plausible that nasopharyngeal carriage of *Pseudomonas* may occur in Fiji.

A key strength of this study is that samples were obtained from a randomised control trial in a setting where PCV has not been introduced. Additionally, PCV7 was not available on the private market when the samples were collected, therefore herd effects are unlikely to impact unvaccinated participants in our study. This means that participants would not be subject to any indirect effects of PCV, and samples were collected during the same time period and from children in a similar setting. Limitations are that the samples were tested following approximately 10 years of storage at − 80 °C, that PCV7 has been replaced by higher valency vaccines (PCV10 and PCV13 are currently in use globally) and that only a single time point was tested. Long-term frozen storage may impact the quality and quantity of DNA recovered from swab samples and has the potential to have an effect on the microbial community structure [57]. However, any effects related to storage are likely to be consistent between groups and therefore would not affect overall conclusions. One limitation of our study is the relatively small sample size, which was partly due to ensuring that there were even numbers of children from each ethnic group represented. Microbiota were not examined at baseline, and groups were not randomised by baseline microbiota. Given the small sample size, it is possible that baseline microbiota may have varied between groups. As such, this study was focussed on exploring associations between PCV7 and nasopharyngeal microbiota rather than inferring direct causality. Given the effect that a small sample may have on some analyses, and potential issues of multiplicity, results should be interpreted with caution. Although PCV7 affects fewer serotypes as PCVs with higher valency, the mechanism of protection is the same; therefore, any trends observed are likely to be similar to those observed for PCV10 or PCV13.

## Conclusion

Overall, we found no significant wider association between PCV and nasopharyngeal microbiota. These results suggest that the majority of replacement that occurs following the elimination of vaccine type pneumococci from the nasopharynx is due to non-vaccine type pneumococci, rather than other bacterial species. However, we found distinct microbial profiles by ethnic group, as well as evidence that the associations with pneumococcal vaccination varied between ethnic groups. Pneumococcal vaccination reduced the relative abundance of *Streptococcus* and *Haemophilus* in iTaukei children and increased the relative abundance of *Dolosigranulum* in FID children. Given the association of *Streptococcus* and *Haemophilus* with URTIs and the association of *Dolosigranulum* with a healthy microbiome, we found that pneumococcal vaccination had positive associations with microbiota in both ethnic groups.

## Methods

### Study design

The swabs used in this study were collected as part of the FiPP, a phase II vaccine trial in Suva, Fiji [26–29]. Details of selection criteria, randomisation procedures and results of the vaccine trial are reported elsewhere [26–29] with key details of trial design found in Russell et al. [27]. In brief, healthy infants were recruited from three participating health centres and were stratified by ethnicity and randomised into groups using a computer-generated list. Participant information was collected by nurses at enrolment (and at swab collection) from the parent/guardian; this included demographic information (e.g. date of birth, sex and ethnicity) and information on known risk factors for pneumococcal colonisation (e.g. exposure to household cigarette smoke, antibiotic use in the previous two weeks and symptoms of an URTI). In this study, we used a total of 144 nasopharyngeal swabs, 36 randomly selected from each of the four groups (iTaukei and FID children; who were either unvaccinated or vaccinated with three doses of PCV7 at 6, 10 and 14 weeks of age). Swabs were collected between 2005 and 2007 from FiPP participants at 12 months of age (prior to 23-valent pneumococcal polysaccharide vaccine administration) and stored and transported as previously described [26]. In brief, buffered cotton swabs (aluminium shaft-buffered; Sarstedt, Australia) were used to sample the nasopharynx for 5 s, before being placed in a sterile cryovial (Simport, Canada) containing 1 ml of skim milk tryptone glucose glycerol (STGG) medium. After chilled transport to the Colonial War Memorial Hospital in Suva, Fiji, the swabs were stored at − 70 °C. Swabs were transported on dry ice to the Pneumococcal Research Laboratory at MCRI in Melbourne, Australia, and stored at − 80 °C.

### DNA extractions

DNA extractions were performed in batches of 23 samples (total of seven batches) plus a negative control (empty sterile 1.5-ml microfuge tube) using the QIamp DNA Mini Kit (Qiagen). In a biohazard class II cabinet, 200 μl of swab STGG was aliquoted into a sterile 1.5-ml microfuge tube for each sample. These aliquots were then spun in a centrifuge for 5 min at 6000×*g* to create a pellet. Pellets were placed at − 80 °C for 15 min to promote cell lysis. Following the freeze/thaw step, 200 μl of enzymatic lysis buffer (containing 36.25 mM phosphate buffer, 1 mg/ml lysozyme, 0.075 mg/ml mutanolysin and 2 mg/ml proteinase K) was added, followed by incubation at 56 °C for 45 min. An additional 20 μl of proteinase K (20 mg/ml, Qiagen) was added before further incubation at 56 °C for 10 min. 4 μl of RNase A (at 100 μg/ml, Qiagen) was added and mixed gently at room temperature for 2 min before adding 200 μl of buffer AL. Samples were then incubated at 70 °C for a further 10 min to complete cell lysis. Extractions were then performed according to the manufacturer’s instructions with DNA eluted in 100 μl of Buffer AE and DNA stored at − 30 °C.

### 16S rRNA gene PCR

The V4 region of the 16S rRNA gene was amplified by PCR using a primer pair which included the Illumina-specific adapter sequences (Table 4). Each PCR was performed using 1 μM of each primer (HPLC purified in liquid at 100 μM concentration, Sigma-Aldrich, Australia), 1X Phusion Green Hot Start II High-Fidelity PCR Master Mix (Thermo Fisher Scientific) and 10 μl of extracted gDNA in a 50-μl reaction. Each PCR run included a no template control which was sequenced along with extraction controls. Following an initial denaturation step at 98 °C for 30 s, there were 35 cycles of denaturation at 98 °C for 5 s, annealing at 65 °C for 20 s and elongation at 72 °C for 15 s, followed by a final elongation step at 72 °C for 5 min. PCR products were then stored at − 20 °C. PCR products from the V4 PCR were purified using AMPure XP beads and following the Illumina ‘16S Metagenomic Sequencing Library Preparation’ protocol (Part # 15044223 Rev. B). PCR product sizes and concentrations were measured by running samples on a TapeStation System (Agilent Technologies) using the D1000 ScreenTape System (Agilent Technologies). MiSeq sequencing was performed by the Translational Genomics Unit at MCRI, Melbourne, Australia. Initially, a sequencing run of 22 samples (plus the extraction control and the no template PCR control) was performed, with the remaining samples and controls then sequenced in two batches. As the sequencing depth varied between the initial sequencing run and the two batches performed later, four of the initial 22 samples were also included in the later sequencing batches to check results that did not differ by sequencing depth.

**Table 4:** 16S rRNA PCR primers for V4 region plus illumina adapter

Region	Product size (bp)*	Primer	Sequence (5′ > 3′)
V4	~ 250	515F	TCGTCGGCAGCGTCAGATGTGTATAAGAGACAG GTGCCAGCMGCCGCGGTAA
		806R	GTCTCGTGGGCTCGGAGATGTGTATAAGAGACAG GGACTACHVGGGTWTCTAAT

*Exact product size varies between bacterial species. Illumina adapter sequence is underlined

The V4 purified DNA was prepared for sequencing using the Illumina ‘16S Metagenomic Sequencing Library Preparation’ protocol (Document # 15044223 Rev. B). The libraries were sequenced using Illumina MiSeq V3 reagent kits (2 × 300 bp) on the MiSeq platform (Illumina).

### Sequence processing

Raw sequences were processed and classified using MOTHUR (version 1.35.1) [58] following the MiSeq SOP (http://www.mothur.org/wiki/MiSeq_SOP; accessed 27 July, 2015) and Silva nr (v119) classification database [59]. Sequences with read lengths falling outside a 225–325 base pairs (bp) range, any ambiguous bases and > 8 bp homopolymers were removed. Sequences were aligned across the region spanning the forward and reverse primers, and any overhang was trimmed. Chimeric sequences were identified and removed using UCHIME [60] and sequences were clustered into OTUs at 97% similarity to the genus level. OTUs for which > 33% of sequence reads were from controls were removed from count data to reduce background signal. Samples which had less than 50,000 sequence reads were removed from further analysis, as were any samples that shared greater than 20% identity (based on relative abundance) to the controls (both extraction and PCR) in downstream analysis as these were considered to have amplified little, if any, bacterial DNA from the swab.

### Species-specific qPCR

Species-specific qPCR assays were used to detect, and determine the carriage densities, of the respiratory pathogens *S. pneumoniae*, *Staphylococcus aureus*, *Moraxella catarrhalis* and *Haemophilus influenzae* [25]. Two duplex assays were performed using primers (Sigma-Aldrich), probes (Eurogentec) and concentrations as described previously [30]. Samples were run in duplicate wells using 2 μl of extracted DNA and Brilliant III Ultra-Fast qPCR Master Mix (Agilent Technologies). Standard curves for each species were prepared using a dilution series of DNA extracted from *S. pneumoniae* ATCC 6305, *H. influenzae* F412, *M. catarrhalis* ATCC 8176 and *S. aureus* ATCC 29213. Duplex qPCRs were performed on a Stratagene Mx3005 instrument with 40 cycles of 95 °C for 20 s and 60 °C for 20 s after an initial activation of 95 °C for 3 min. Results were analysed using MxPro™ software (Stratagene) and genomic equivalents per millilitre determined for each sample.

### Pseudomonas culture

Eight samples (FM052, FM108, FM029, FM001, FM004, FM129, FM057, FM020) which contained *Pseudomonas* at a relative abundance > 90% were cultured on cephaloridine fucidin cetrimide (CFC) agar (Oxoid, Thermo Fisher) at 28 °C for 48 h. Isolated colonies were then confirmed as Gram-negative bacilli that were oxidase positive and identified by MALDI-TOF MS using the Vitek® MS system (bioMérieux).

### Analyses

Analyses were conducted and graphs were generated using GraphPad Prism version 7.02 unless otherwise stated. *P* < 0.05 was considered statistically significant. Relative abundance was defined as the percentage of total sequence reads from an individual sample that were from the specific OTU, genus or family being considered. Relative abundance (%) data was used as input for analyses unless otherwise stated.

For comparing carriage prevalence from the species-specific qPCR data, Fisher’s exact test was used. For comparing carriage densities from the species-specific qPCR, data were first checked for normality using the D’Agostino & Pearson normality test. For normally distributed data, an unpaired *t* test was used. Where data were not normally distributed, the Mann-Whitney test was used.

Alpha-diversity (i.e. within sample diversity) was determined through OTU richness (number of OTUs) and Shannon diversity index. Alpha-diversity measures were calculated using R Studio (version 3.2.4) and the ‘diversity’ function of the vegan package (version 2.3-5) [61]. Richness calculations were rarefied to account for differences in sequencing depth, as greater sequencing depth increases the number of OTUs or richness. Rarefaction was done by subsampling 50,000 sequence reads (based on the lowest number of reads—53,616 reads) using the ‘rarefy’ function in vegan package. Beta-diversity (i.e. between sample diversity) was calculated using a Jaccard distance matrix (‘vegdist’ function, R vegan package) using both abundance-based (default setting) and binary-based (binary = TRUE) measures with nMDS plots (‘metaMDS’ function, vegan package) as the ordination method. Permutational multivariate analysis of variance (PERMANOVA, ‘adonis’ function, R vegan package) was used to calculate significant differences in microbial composition between groups.

A heatmap of microbial composition in all samples was generated in R using the following packages: vegan, RColorBrewer (version 1.1-2), gplots (version 3.0.1) and dendextend (version 1.7.0). Samples and OTUs were clustered using the ‘hclust’ function and the average linkage method based on a Jaccard distance matrix. For generating the heatmap, OTUs were filtered to only those with a relative abundance above 30% in at least one sample. Clusters were characterised using the ‘cascadeKM’ function (vegan package) with the default ‘calinski’ criterion (Calinski-Harabasz [62]) and coloured using the ‘color_branches’ function (dendextend package).

To examine co-presence or mutual-exclusion of the most abundant OTUs, a correlation network was generated using count data and the default SPARCC [63] parameters in MOTHUR after filtering to the top 100 most abundant OTUs. The SPARCC data was then formatted and analysed in R. Only correlations with a significance of *p* < 0.05, a correlation *r* > 0.3 and OTUs with a classification to the genus level were included in the network. Networks were visualised and edited using Cytoscape software version 3.3.0 [64].

Random forest models were used for each risk factor to rank OTUs on their ability to discriminate between groups (see Additional file 4: Table S4). The random forest analysis was done in RStudio using R packages ‘randomForest’ version 4.6-14, ‘pylr’ version 1.8.4 and ‘rfUtilities’ version 2.1-3. Using relative abundance data, rare OTUs that were zero in greater than 95% of samples were removed, data were then normalised back to 100%. Each risk factor was analysed in separate models using the ‘randomForest’ and ‘rf.significance’ functions. Each model used the parameters ‘ntree = 501’, ‘importance = TRUE’ and ‘proximities = TRUE’, and significance was calculated using 500 permutations. The mean decrease in accuracy or variable importance was examined in each model for the top 10 OTUs.

Multivariable linear regression (Additional file 5 and Additional file 6) was done in RStudio using the ‘lm’ function (base ‘stats’ package in R, version 3.2.4) for looking at specific associations with vaccination and ethnicity on pooled genus data or α-diversity measures. For each model, an interaction term between vaccination and ethnicity was considered and Akaike information criterion was used to determine whether inclusion of the interaction term was appropriate. As well as vaccination status and ethnicity, final models included the following risk factors: symptoms of an URTI (such as runny nose or cough), breastfeeding status, household exposure to cigarette smoke, the year the swab was collected, season of swab collection, antibiotic use in the previous two weeks and sex of the child. Each model also included the sequence count for each sample to account for potential differences in sequencing depth between samples. Checks of the normality of residuals were performed using the ‘plot’ function in R, in cases where residuals were not normal, data were log transformed.

## Additional files

Additional file 1: Participant characteristics. Participant characteristics stratified by ethnicity (**Table S1.**) and by vaccination status (**Table S2.**). As well as participant characteristics for those included in this study vs. those not included (**Table S3.**). (DOCX 22 kb)

Additional file 2: Richness and Shannon diversity. Plots of richness and Shannon diversity index (**Figure S1.**) by vaccination status (a and b), by ethnicity (c and d) and by vaccination status within each ethnic group (e and f). (DOCX 674 kb)

Additional file 3: Species-specific qPCR data. Species-specific qPCR results (carriage prevalence and density) for *S. pneumoniae*, *H. influenzae*, *M. catarrhalis* and *S.* *aureus* by vaccination status (**Figure S2.**), ethnicity (**Figure S3.**) and vaccination status after stratifying by ethnicity (**Figure S4.**). (DOCX 954 kb)

Additional file 4: Association between microbial composition and participant characteristics by PERMANOVA and random forest models. Table (**Table S4.**) of the PERMANOVA and random forest results for each of the participant characteristics. (DOCX 19 kb)

Additional file 5: URTI symptoms and ethnicity. Table (**Table S5.**) of the median relative abundance (%) for the seven most common genera by symptoms of an upper respiratory tract infection (URTI) after stratifying by ethnicity. (DOCX 15 kb)

Additional file 6: Linear regression data. Unadjusted (**Table S6.**) and adjusted (**Table S7.**) linear regression results, as well as this data following log transformation (**Tables S8** and **S9.**) for models that were log transformed. (DOCX 28 kb)

## Data Availability

The datasets generated and analysed during the current study are available in the NCBI Sequence Read Archive, accession number PRJNA493513 (https://www.ncbi.nlm.nih.gov/sra/PRJNA493513).

## Abbreviations

FID: Fijians of Indian descent
FiPP: Fiji Pneumococcal Project
nMDS: Non-metric multidimensional scaling
OTU: Operational taxonomic unit
PCV: Pneumococcal conjugate vaccine
qPCR: Real-time quantitative PCR
URTI: Upper respiratory tract infection
