# Supplementary material for: The association between pneumococcal vaccination, ethnicity, and the nasopharyngeal microbiota of children in Fiji
Source: Microbiome. 2019 Jul 16;7:106. doi: 10.1186/s40168-019-0716-4 (PMC6636143; doi:10.1186/s40168-019-0716-4)
Supplement: Supplementary file 1 — Participant characteristics. Participant characteristics stratified by ethnicity (Table S1.) and by vaccination status (Table S2.). As well as participant characteristics for those included in this study vs. those not included (Table S3.). (DOCX 22 kb) [file 40168_2019_716_MOESM1_ESM.docx]

Table S1. Participant characteristics for children aged 12 months by ethnicity.

| **Characteristics at swab collection** | **iTaukei**  (n=67) | **FID**  (n=65) | **p-value** |
| --- | --- | --- | --- |
| Male, n (%) | 37 (55) | 34 (52) | 0.862 |
| Swab collected in wet season^1^, n (%) | 53 (79) | 45 (69) | 0.234 |
| Mean weight^2^ in g (SD) | *5023 (578)* | *4382 (526)* | *<0.001* |
| Breastfeeding, n (%) | 45 (67) | 39 (60) | 0.470 |
| Median age breastfeeding stopped^3^ in wks (IQR) | 27 (22-36) | 23 (9-34) | 0.104 |
| Exposure to cigarette smoking, n (%) | *37 (55)* | *12 (18)* | *0.038* |
| Prior antimicrobial use^4^, n (%) | 9 (13) | 0 (0) | 0.243 |
| Symptoms of URTI [any], n (%) | 25 (37) | 14 (22) | 0.057 |
| Runny nose, n (%) | 19 (28) | 11 (17) | 0.147 |
| Cough, n (%) | 17 (25) | 9 (14) | 0.126 |

URTI, upper respiratory tract infection; SD, standard deviation; iTaukei, indigenous Fijian; FID, Fijian of Indian Descent. Statistically significant differences (p<0.05) as calculated by Fisher’s Exact test are shown in italics. ^1^Wet season = November – April; ^2^Weight data available for 46/67 iTaukei children and 56/65 FID children; ^3^Data only included for children that had stopped breastfeeding; ^4^Antimicrobial use in the prior two weeks as reported by parent/guardian.

Table S2. Participant characteristics for children aged 12 months by vaccination status.

| **Characteristics at swab collection** | **No PCV7**  (n=65) | **PCV7**  (n=67) | **p-value** |
| --- | --- | --- | --- |
| Male, n (%) | 30 (46) | 41 (61) | 0.116 |
| Swab collected in wet season^1^, n (%) | 50 (77) | 48 (72) | 0.553 |
| Mean weight^2^ in g (SD) | 4627 (679) | 4748 (548) | 0.328 |
| Breastfeeding, n (%) | *48 (74)* | *36 (54)* | *0.019* |
| Median age breastfeeding stopped^3^ in wks (IQR) | 26 (8-34) | 28 (16-38) | 0.280 |
| Exposure to cigarette smoking, n (%) | 33 (51) | 28 (42) | 0.383 |
| Prior antimicrobial use^4^, n (%) | 8 (12) | 5 (7) | 0.394 |
| Symptoms of URTI [any], n (%) | 19 (29) | 20 (30) | 1.000 |
| Runny nose, n (%) | 16 (25) | 14 (21) | 0.680 |
| Cough, n (%) | 11 (17) | 15 (22) | 0.514 |

URTI, upper respiratory tract infection; SD, standard deviation. Statistically significant differences (p<0.05) as calculated by Fisher’s Exact test are shown in italics. ^1^Wet season = November – April; ^2^Weight data available for 37/67 vaccinated children and all unvaccinated children; ^3^Data only included for children that had stopped breastfeeding; ^4^Antimicrobial use in the prior two weeks as reported by parent/guardian.

Table S3. Participant characteristics for children aged 12 months involved in the FiPP that were included and not included in the current study.

| **Characteristics at swab collection** | **Included in current study** | | | |  | **Not included in current study** | | | |
| --- | --- | --- | --- | --- | --- | --- | --- | --- | --- |
|  | **No PCV7 iTaukei** (n=33) | **PCV7 iTaukei** (n=34) | **No PCV7 FID** (n=32) | **PCV7 FID**  (n=33) |  | **No PCV7 iTaukei**  (n=43) | **PCV7 iTaukei** (n=35) | **No PCV7 FID**  (n=6) | **PCV7**  **FID**  (n=5) |
| Male, n (%) | 18 (55) | 19 (56) | 12 (38) | 22 (67) |  | 18 (42) | 16 (46) | 4 (67) | 3 (60) |
| Swab collected in wet season^1^, n (%) | *25 (76)*^5^ | *28 (82)*^5^ | 25 (78) | 20 (61) |  | *23 (53)*^5^ | *22 (63)*^5^ | 4 (67) | 4 (80) |
| Mean weight^2^ in g (SD) | 4998 (584) | 5084 (579) | 4248 (547) | 4566 (443) |  | 5007 (656) | 5127 (746) | 4430 (436) | 4747 (656) |
| Breastfeeding, n (%) | 26 (79) | 19 (56) | 22 (69) | 17 (52) |  | 28 (65) | 19 (54) | 5 (83) | 1 (20) |
| Median age breastfeeding stopped^3^ in wks (IQR) | 30 (26-46) | 28 (18-35) | 9 (2-31) | 27 (15-40) |  | 26 (16-40) | 39 (31-44) | 8 | 38 (32-41) |
| Exposure to cigarette smoking, n (%) | 21 (64) | 16 (47) | 12 (38) | 12 (36) |  | 23 (53) | 19 (54) | 2 (33) | 3 (60) |
| Prior antimicrobial use^4^, n (%) | 4 (12) | 5 (15) | 4 (13) | 0 (0) |  | 2 (5) | 6 (17) | 0 (0) | 0 (0) |
| Symptoms of URTI [any], n (%) | 11 (33) | 14 (41) | 8 (25) | 6 (18) |  | 19 (44) | 15 (43) | 0 (0) | 0 (0) |
| Runny nose, n (%) | 10 (30) | 9 (26) | 6 (19) | 5 (15) |  | 10 (23) | 11 (31) | 0 (0) | 0 (0) |
| Cough, n (%) | 7 (21) | 10 (29) | 4 (13) | 5 (15) |  | 14 (33) | 10 (29) | 0 (0) | 0 (0) |

URTI, upper respiratory tract infection; SD, standard deviation; IQR, interquartile range; iTaukei, indigenous Fijian; FID, Fijian of Indian Descent. Statistically significant differences (p<0.05) as calculated by Fisher’s Exact test are shown in italics. ^1^Wet season = November – April; ^2^Weight data available for 46/67 iTaukei children and 56/65 FID children; ^3^Data only included for children that had stopped breastfeeding; ^4^Antimicrobial use in the prior two weeks as reported by parent/guardian; ^5^Significantly more iTaukei children included in the study had swabs collected in the wet season compared with iTaukei children not included in the study (79% vs. 58%).
