# Supplementary material for: The association between pneumococcal vaccination, ethnicity, and the nasopharyngeal microbiota of children in Fiji
Source: Microbiome. 2019 Jul 16;7:106. doi: 10.1186/s40168-019-0716-4 (PMC6636143; doi:10.1186/s40168-019-0716-4)
Supplement: Supplementary file 2 — Richness and Shannon diversity. Plots of richness and Shannon diversity index (Figure S1.) by vaccination status (a and b), by ethnicity (c and d) and by vaccination status within each ethnic group (e and f). (DOCX 674 kb) [file 40168_2019_716_MOESM2_ESM.docx]

**d**

**b**

**a**

**c**

**e**

**f**

Figure S1. Richness and Shannon diversity index by vaccination status (a and b), by ethnicity (c and d) and by vaccination status within each ethnic group (e and f). Error bars are median and interquartile range. Only significant differences (p<0.05) are shown.
