# Supplementary material for: The association between pneumococcal vaccination, ethnicity, and the nasopharyngeal microbiota of children in Fiji
Source: Microbiome. 2019 Jul 16;7:106. doi: 10.1186/s40168-019-0716-4 (PMC6636143; doi:10.1186/s40168-019-0716-4)
Supplement: Supplementary file 3 — Species-specific qPCR data. Species-specific qPCR results (carriage prevalence and density) for S. pneumoniae, H. influenzae, M. catarrhalis and S. aureus by vaccination status (Figure S2.), ethnicity (Figure S3.) and vaccination status after stratifying by ethnicity (Figure S4.). (DOCX 954 kb) [file 40168_2019_716_MOESM3_ESM.docx]

**b**

**a**

Figure S2. Carriage prevalence (a) and density (b) of *S. pneumoniae*, *H. influenzae*, *M. catarrhalis* and *S.* *aureus* (as determined by qPCR) by vaccination status. Error bars are (a) 95% confidence intervals and (b) median and interquartile range. No significant differences were observed.

**a**

**b**

Figure S3. Carriage prevalence (a) and density (b) of *S. pneumoniae*, *H. influenzae*, *M. catarrhalis* and *S.* *aureus* (as determined by qPCR) by ethnicity. Error bars are (a) 95% confidence intervals and (b) median and interquartile range. Significant differences were observed in carriage prevalence of *S. pneumoniae*, *H. influenzae* and *M. catarrhalis* between iTaukei and FID children.

 Figure S4. Carriage prevalence (a) and density (b) of *S. pneumoniae*, *H. influenzae*, *M. catarrhalis* and *S.* *aureus* (as determined by qPCR) by vaccination status within each ethnic group. Error bars are (a) 95% confidence intervals and (b) median and interquartile range. No significant differences were observed by vaccination status within each ethnic group.

**b**

**a**
