# Supplementary material for: The association between pneumococcal vaccination, ethnicity, and the nasopharyngeal microbiota of children in Fiji
Source: Microbiome. 2019 Jul 16;7:106. doi: 10.1186/s40168-019-0716-4 (PMC6636143; doi:10.1186/s40168-019-0716-4)
Supplement: Supplementary file 4 — Association between microbial composition and participant characteristics by PERMANOVA and random forest models. Table (Table S4.) of the PERMANOVA and random forest results for each of the participant characteristics. (DOCX 19 kb) [file 40168_2019_716_MOESM4_ESM.docx]

Table S4. Association between microbial composition and participant characteristics using PERMANOVA (adonis) and random forest models.

|  | **PERMANOVA** | | |  | **Random forest** | |
| --- | --- | --- | --- | --- | --- | --- |
| **Parameter** | **F value** | **R^2^** | **p-value** |  | **Model OOB Error (%)** | **p-value** |
| Ethnicity of the child | *5.456* | *0.04* | *<0.001* |  | *33* | *<0.001* |
| Symptoms of an URTI (any) | *3.465* | *0.026* | *0.003* |  | *29* | *0.012* |
| runny nose | *4.091* | *0.031* | *0.002* |  | *20* | *0.004* |
| cough | *2.515* | *0.019* | *0.026* |  | 20 | 0.058 |
| Season of swab collection^1^ | 1.638 | 0.012 | 0.106 |  | 32 | 0.480 |
| Exposure to cigarette smoking | 1.205 | 0.009 | 0.271 |  | 45 | 0.238 |
| Year of swab collection | 1.095 | 0.017 | 0.329 |  | 60 | 0.504 |
| Breastfeeding | 1.015 | 0.008 | 0.383 |  | 45 | 0.570 |
| Prior antimicrobial use^2^ | 0.723 | 0.006 | 0.667 |  | 11 | 0.358 |
| Vaccination status | 0.702 | 0.005 | 0.668 |  | 58 | 0.836 |
| Sex of the child | 0.533 | 0.004 | 0.838 |  | 55 | 0.886 |

URTI, upper respiratory tract infection; OOB, out-of-bag. Statistically significant differences (p<0.05) are shown in italics. ^1^Wet season = November – April, Dry season = May – October; ^2^Antimicrobial use in the prior two weeks as reported by parent/guardian.
