# Supplementary material for: The association between pneumococcal vaccination, ethnicity, and the nasopharyngeal microbiota of children in Fiji
Source: Microbiome. 2019 Jul 16;7:106. doi: 10.1186/s40168-019-0716-4 (PMC6636143; doi:10.1186/s40168-019-0716-4)
Supplement: Supplementary file 5 — URTI symptoms and ethnicity. Table (Table S5.) of the median relative abundance (%) for the seven most common genera by symptoms of an upper respiratory tract infection (URTI) after stratifying by ethnicity. (DOCX 15 kb) [file 40168_2019_716_MOESM5_ESM.docx]

Table S5. Median relative abundance (%) for the seven most common genera by symptoms of an upper respiratory tract infection (URTI) after stratifying by ethnicity.

|  | **iTaukei** | | **p-value^1^** | **FID** | | **p-value^1^** |
| --- | --- | --- | --- | --- | --- | --- |
|  | **no URTI**  (n=42) | **URTI**  (n=25) |  | **no URTI**  (n=51) | **URTI**  (n=14) |  |
| *Pseudomonas*^2^ | 0.04  (0.01-3.63) | 0.03  (0.01-0.12) | 0.591 | 0.03  (0.01-0.28) | 0.06  (0.02-0.16) | 0.744 |
| *Moraxella* | 29.01  (1.53-45.81) | 32.80  (20.15-47.10) | 0.995 | 0.09  (0.05-28.47) | 0.90  (0.14-48.18) | 0.110 |
| *Staphylococcus*^2^ | 0.02  (0.01-0.05) | 0.03  (0.01-0.07) | 0.728 | 0.07  (0.03-0.26) | 0.02  (0.01-0.73) | 0.460 |
| *Dolosigranulum* | 21.99  (7.15-36.22) | 10.30  (4.88-28.85) | 0.157 | 44.59  (18.83-51.61) | 25.19  (1.77-33.80) | 0.219 |
| *Streptococcus*^2^ | 0.81  (0.27-3.49) | 1.57  (0.19-6.25) | 0.555 | 0.35  (0.11-3.41) | 1.32  (0.73-8.90) | 0.094 |
| *Corynebacterium* | 7.76  (2.05-17.89) | 1.49  (0.80-10.38) | 0.456 | 24.05  (4.96-44.39) | 3.72  (2.09-19.43) | 0.082 |
| *Haemophilus*^2^ | *0.08*  *(0.02-3.70)* | *6.85*  *(0.16-23.57)* | *0.004* | 0.03  (0.01-0.27) | 0.14  (0.01-1.96) | 0.546 |

URTI, upper respiratory tract infection; iTaukei, indigenous Fijian; FID, Fijian of Indian Descent. Data are median (interquartile range). Statistically significant differences are shown in italics. ^1^p-value calculated following multivariate linear regression adjusting for ethnicity, symptoms of an upper respiratory tract infection, exposure to household cigarette smoke, breastfeeding status, year of swab collection, season of swab collection, antibiotic use in the previous two weeks and sex of the child. An interaction between vaccination status and ethnicity was included in all models except those for *Moraxella* and *Staphylococcus*; ^2^Log transformation of relative abundance was used in the linear regression models.
