# Supplementary material for: The association between pneumococcal vaccination, ethnicity, and the nasopharyngeal microbiota of children in Fiji
Source: Microbiome. 2019 Jul 16;7:106. doi: 10.1186/s40168-019-0716-4 (PMC6636143; doi:10.1186/s40168-019-0716-4)
Supplement: Supplementary file 6 — Linear regression data. Unadjusted (Table S6.) and adjusted (Table S7.) linear regression results, as well as this data following log transformation (Tables S8 and S9.) for models that were log transformed. (DOCX 28 kb) [file 40168_2019_716_MOESM6_ESM.docx]

Table S6. Unadjusted and untransformed linear regression data for the seven most common genera by vaccination status (overall), by ethnicity, and by vaccination status within each ethnic group.

|  | Vaccination | | | Ethnicity | | | iTaukei Vaccination | | | FID Vaccination | | |
| --- | --- | --- | --- | --- | --- | --- | --- | --- | --- | --- | --- | --- |
|  | **Coeff.** | **95% CI** | **p-value** | **Coeff.** | **95% CI** | **p-value** | **Coeff.** | **95% CI** | **p-value** | **Coeff.** | **95% CI** | **p-value** |
| *Pseudomonas* | -2.05 | -12.65, 8.56 | 0.703 | -5.58 | -16.15, 4.98 | 0.298 | 11.03 | -5.17, 27.23 | 0.179 | *-15.52* | *-28.74, -2.30* | *0.022* |
| *Moraxella* | 0.38 | -8.27, 9.02 | 0.932 | *-13.22* | *-21.56, -4.88* | *0.002* | 1.68 | -10.38, 13.74 | 0.782 | -0.96 | -12.89, 10.96 | 0.872 |
| *Staphylococcus* | 1.18 | -0.93, 3.29 | 0.271 | 0.15 | -1.97, 2.27 | 0.888 | 0.99 | -1.85, 3.83 | 0.489 | 1.37 | -1.86, 4.60 | 0.399 |
| *Dolosigranulum* | 4.85 | -2.57, 12.27 | 0.198 | *12.98* | *5.86, 20.09* | *p<0.001* | -1.86 | -10.82, 7.10 | 0.679 | *11.76* | *0.72, 22.81* | *0.037* |
| *Streptococcus* | *-5.00* | *-8.48, -1.53* | *0.005* | 0.29 | -3.29, 3.87 | 0.872 | *-5.60* | *-9.69, -1.50* | *0.008* | -4.39 | -10.19, 1.41 | 0.135 |
| *Corynebacterium* | 0.84 | -5.44, 7.13 | 0.792 | *10.91* | *4.91, 16.91* | *p<0.001* | -2.44 | -9.28, 4.39 | 0.478 | 4.22 | -5.90, 14.34 | 0.407 |
| *Haemophilus* | 0.29 | -6.20, 6.79 | 0.929 | -2.70 | -9.17, 3.78 | 0.412 | -4.65 | -13.42, 4.12 | 0.293 | 5.39 | -4.32, 15.10 | 0.271 |

Coeff., coefficient; CI, confidence interval; iTaukei, indigenous Fijian; FID, Fijian of Indian Descent. Statistically significant values (p<0.05) are shown in italics.

Table S7. Adjusted and untransformed linear regression data for the seven most common genera by vaccination status (overall), by ethnicity, and by vaccination status within each ethnic group.

|  | Vaccination | | | Ethnicity | | | iTaukei Vaccination | | | FID Vaccination | | |
| --- | --- | --- | --- | --- | --- | --- | --- | --- | --- | --- | --- | --- |
|  | **Coeff.** | **95% CI** | **p-value** | **Coeff.** | **95% CI** | **p-value** | **Coeff.** | **95% CI** | **p-value** | **Coeff.** | **95% CI** | **p-value** |
| *Pseudomonas* | 14.00 | -0.99, 28.99 | 0.067 | -11.08 | -26.59, 4.43 | 0.160 | 14.47 | -2.78, 31.73 | 0.099 | *-15.77* | *-31.00, -0.55* | *0.043* |
| *Moraxella* | 1.00 | -7.81, 9.81 | 0.823 | *-13.08* | *-21.97, -4.18* | *0.004* | -0.57 | -13.02, 13.19 | 0.927 | -2.69 | -16.14, 10.77 | 0.690 |
| *Staphylococcus* | 0.82 | -1.42, 3.08 | 0.469 | 0.09 | -2.18, 2.35 | 0.941 | 0.51 | -2.48, 3.50 | 0.735 | 0.57 | -3.03, 4.18 | 0.752 |
| *Dolosigranulum* | -1.43 | -11.36, 8.51 | 0.777 | *11.03* | *3.66, 18.41* | *0.004* | -2.24 | -11.54, 7.07 | 0.632 | *13.14* | *0.85, 25.43* | *0.037* |
| *Streptococcus* | *-6.35* | *-11.38, -1.32* | *0.014* | 1.12 | -4.08, 6.33 | 0.670 | *-5.98* | *-10.00, -1.97* | *0.004* | -4.33 | -11.25, 2.59 | 0.216 |
| *Corynebacterium* | 0.34 | -5.98, 6.65 | 0.916 | *9.89* | *3.52, 16.27* | *0.003* | -1.26 | -8.66, 6.14 | 0.735 | 5.67 | -5.51, 16.85 | 0.314 |
| *Haemophilus* | -6.41 | -15.60, 2.78 | 0.170 | -7.35 | -16.85, 2.78 | 0.129 | -6.27 | -14.75, 2.21 | 0.144 | 4.95 | -6.56, 16.46 | 0.392 |

Coeff., coefficient; CI, confidence interval; iTaukei, indigenous Fijian; FID, Fijian of Indian Descent. Statistically significant values (p<0.05) are shown in italics.

Table S8. Unadjusted and log transformed linear regression data for the seven most common genera by vaccination status (overall), by ethnicity, and by vaccination status within each ethnic group.

|  | Vaccination | | | Ethnicity | | | iTaukei Vaccination | | | FID Vaccination | | |
| --- | --- | --- | --- | --- | --- | --- | --- | --- | --- | --- | --- | --- |
|  | **Coeff.** | **95% CI** | **p-value** | **Coeff.** | **95% CI** | **p-value** | **Coeff.** | **95% CI** | **p-value** | **Coeff.** | **95% CI** | **p-value** |
| *Pseudomonas* | -0.37 | -1.50, 0.76 | 0.521 | -0.11 | -1.24, 1.02 | 0.850 | 0.70 | -1.09, 2.49 | 0.437 | *-1.45* | *-2.84, -0.06* | *0.041* |
| *Staphylococcus* | 0.10 | -0.62, 0.82 | 0.789 | *0.91* | *0.20, 1.60* | *0.012* | -0.10 | -1.14, 0.95 | 0.853 | 0.28 | -0.69, 1.25 | 0.564 |
| *Streptococcus* | -0.58 | -1.34, 0.18 | 0.134 | -0.31 | -1.08, 0.45 | 0.418 | *-1.16* | *-2.19, -0.12* | *0.029* | 0.02 | -1.11, 1.14 | 0.978 |
| *Haemophilus* | -0.42 | -1.57, 0.72 | 0.463 | *-1.55* | *-2.66, -0.43* | *0.007* | -1.30 | -2.88, 0.29 | 0.107 | 0.50 | -1.08, 2.08 | 0.526 |

Coeff., coefficient; CI, confidence interval; iTaukei, indigenous Fijian; FID, Fijian of Indian Descent. Statistically significant values (p<0.05) are shown in italics.

Table S9. Adjusted and log transformed linear regression data for the seven most common genera by vaccination status (overall), by ethnicity, and by vaccination status within each ethnic group.

|  | Vaccination | | | Ethnicity | | | iTaukei Vaccination | | | FID Vaccination | | |
| --- | --- | --- | --- | --- | --- | --- | --- | --- | --- | --- | --- | --- |
|  | **Coeff.** | **95% CI** | **p-value** | **Coeff.** | **95% CI** | **p-value** | **Coeff.** | **95% CI** | **p-value** | **Coeff.** | **95% CI** | **p-value** |
| *Pseudomonas* | 1.03 | -1.50, 0.76 | 0.212 | 1.32 | -0.36, 2.99 | 0.122 | 1.20 | -0.69, 3.09 | 0.208 | -1.35 | -2.93, 0.22 | 0.090 |
| *Staphylococcus* | 0.05 | -0.62, 0.82 | 0.887 | *0.86* | *0.10, 1.61* | *0.026* | -0.19 | -1.35, 0.96 | 0.738 | 0.48 | -0.66, 1.61 | 0.403 |
| *Streptococcus* | *-1.16* | *-1.34, 0.18* | *0.030* | -0.95 | -2.04, 0.13 | 0.085 | *-1.21* | *-2.24, -0.18* | *0.022* | 0.50 | -0.79, 1.79 | 0.438 |
| *Haemophilus* | -1.50 | -1.57, 0.72 | 0.053 | *-2.38* | *-3.97, -0.79* | *0.004* | *-1.52* | *-2.98, -0.05* | *0.043* | 0.71 | -1.21, 2.62 | 0.462 |

Coeff., coefficient; CI, confidence interval; iTaukei, indigenous Fijian; FID, Fijian of Indian Descent. Statistically significant values (p<0.05) are shown in italics.
